# Artificial Intelligence for Clinical Diagnosis and Treatment of Prostate Cancer

**DOI:** 10.3390/cancers14225595

**Published:** 2022-11-14

**Authors:** Ali A. Rabaan, Muhammed A. Bakhrebah, Hajir AlSaihati, Saad Alhumaid, Roua A. Alsubki, Safaa A. Turkistani, Saleh Al-Abdulhadi, Yahya Aldawood, Abdulmonem A. Alsaleh, Yousef N. Alhashem, Jenan A. Almatouq, Ahlam A. Alqatari, Hejji E. Alahmed, Dalal A. Sharbini, Arwa F. Alahmadi, Fatimah Alsalman, Ahmed Alsayyah, Abbas Al Mutair

**Affiliations:** 1 Molecular Diagnostic Laboratory, Johns Hopkins Aramco Healthcare, Dhahran 31311, Saudi Arabia; 2 College of Medicine, Alfaisal University, Riyadh 11533, Saudi Arabia; 3 Department of Public Health and Nutrition, The University of Haripur, Haripur 22610, Pakistan; 4 Life Science and Environment Research Institute, King Abdulaziz City for Science and Technology (KACST), Riyadh 11442, Saudi Arabia; 5 Department of Clinical Laboratory Sciences, College of Applied Medical Sciences, University of Hafr Al Batin, Hafr Al Batin 39831, Saudi Arabia; 6 Administration of Pharmaceutical Care, Al-Ahsa Health Cluster, Ministry of Health, Al-Ahsa 31982, Saudi Arabia; 7 Department of Clinical Laboratory Sciences, College of Applied Medical Sciences, King Saud University, Riyadh 11362, Saudi Arabia; 8 Department of Medical Laboratory Sciences, Fakeeh College for Medical Science, Jeddah 21134, Saudi Arabia; 9 Department of Medical Laboratory Sciences, College of Applied Medical Sciences, Prince Sattam Bin Abdulaziz University, Riyadh 11942, Saudi Arabia; 10 Clinical Laboratory Science Department, Mohammed Al-Mana College for Medical Sciences, Dammam 34222, Saudi Arabia; 11 Hematopathology Department, Clinical Pathology, Al-Dorr Specialist Medical Center, Qatif 31911, Saudi Arabia; 12 Department of Laboratory and Blood Bank, King Fahad Hospital Hofuf, Al Hofuf 36441, Saudi Arabia; 13 Immunology and Serology Laboratory, King Fahd Military Medical Complex Dhahran, Dhahran 31932, Saudi Arabia; 14 Department of Clinical Laboratory Sciences, College of Applied Medical Sciences, Imam Abdulrahman Bin Faisal University, Dammam 31441, Saudi Arabia; 15 Department of Emergency Medicine, Oyun City Hospital, Al-Ahsa 36312, Saudi Arabia; 16 Department of Pathology, College of Medicine, Imam Abdulrahman Bin Faisal University, Dammam 31441, Saudi Arabia; 17 Research Center, Almoosa Specialist Hospital, Al-Ahsa 36342, Saudi Arabia; 18 College of Nursing, Princess Norah Bint Abdulrahman University, Riyadh 11564, Saudi Arabia; 19 School of Nursing, Wollongong University, Wollongong, NSW 2522, Australia; 20 Nursing Department, Prince Sultan Military College of Health Sciences, Dhahran 33048, Saudi Arabia

**Keywords:** artificial intelligence, clinical diagnosis, prostate cancer, machine learning

## Abstract

**Simple Summary:**

The primary purpose of this review is to provide an in-depth analysis of existing Artificial Intelligence (AI) algorithms used in the field of prostate cancer (PC) for diagnosis and treatment. This review aims to show the research community that AI-enabled technologies have the potential for widespread growth and penetration of PC diagnostics and therapeutics to simplify and accelerate existing healthcare processes.

**Abstract:**

As medical science and technology progress towards the era of “big data”, a multi-dimensional dataset pertaining to medical diagnosis and treatment is becoming accessible for mathematical modelling. However, these datasets are frequently inconsistent, noisy, and often characterized by a significant degree of redundancy. Thus, extensive data processing is widely advised to clean the dataset before feeding it into the mathematical model. In this context, Artificial intelligence (AI) techniques, including machine learning (ML) and deep learning (DL) algorithms based on artificial neural networks (ANNs) and their types, are being used to produce a precise and cross-sectional illustration of clinical data. For prostate cancer patients, datasets derived from the prostate-specific antigen (PSA), MRI-guided biopsies, genetic biomarkers, and the Gleason grading are primarily used for diagnosis, risk stratification, and patient monitoring. However, recording diagnoses and further stratifying risks based on such diagnostic data frequently involves much subjectivity. Thus, implementing an AI algorithm on a PC’s diagnostic data can reduce the subjectivity of the process and assist in decision making. In addition, AI is used to cut down the processing time and help with early detection, which provides a superior outcome in critical cases of prostate cancer. Furthermore, this also facilitates offering the service at a lower cost by reducing the amount of human labor. Herein, the prime objective of this review is to provide a deep analysis encompassing the existing AI algorithms that are being deployed in the field of prostate cancer (PC) for diagnosis and treatment. Based on the available literature, AI-powered technology has the potential for extensive growth and penetration in PC diagnosis and treatment to ease and expedite the existing medical process.

## 1. Introduction

Prostate cancer (PC) is the second most commonly diagnosed cancer in the male population and it is the most common cancer type in the United States. In 2020, WHO provided cancer statistics, which showed 1,414,259 cases of PC in the complete dataset [1]. It was also noted that PC is the most common disease among the Afro-American races. As per the National Institute of Health (NIH) data, 268,490 new cases of PC were reported in 2022, with there being 34,500 deaths worldwide [2]. The mortality rate was found to be increased with an individual’s age, thus, it is the most prevalent among individuals over 66 years, accounting for more than 55% of the total number of deaths [1]. It was found that the size of the prostate gland increases with age, which is termed prostatic hyperplasia (BPH). BPH causes symptoms such as frequent urination which is caused by the compression of the bladder due to an enlarged prostate. It affects 33% of men over 60 and 50% of men that are over 80 years old. An earlier study indicated BPH as a precursor for PC, but could not establish a clear association [3].

The associated risk factors for PC included age (above 40) and race (more common in Black or Afro-American races) as evident from the data of the surveillance, epidemiology, and end results (SEER) US population registry [4]. Moreover, other factors, such as genetic mutations in the BRCA2 gene [5], family history [6], smoking [7], obesity, and eating high fat-containing foods [8], were also linked to the onset of PC. Additionally, more risk factors for PC include a history of prostatitis, the inflammation of the prostate gland, and the administration of drugs that inhibit 5 alpha-reductase, which is used to treat BPH [9]. During the initial stage, patients with PC do not exhibit major symptoms, except common complaints regarding the difficulty with urination, frequent urination, and nighttime urination, which resemble those of BPH. Symptoms, such as urine retention and back discomfort are often indicators of the disease. Moreover, back pain is also an indication of the metastatic stage of PC, showing its spread to the bones [10].

In the healthcare industry, digitizing and storing big medical data has critically contributed to applying artificial intelligence-based techniques in diagnosis and management. Artificial intelligence (AI) is an automated computing process with a built-in programmed intelligence that is used to make decisions in an unfamiliar environment. Generally, the term AI is commonly used to describe the robotic processes constituted of computer algorithms that are connected to the machinery hardware. With advancements in AI, including the development of machine learning (ML) algorithms and deep learning (DL) models using mathematical rules and statistical assumptions, the machines are manipulated or trained to understand the hidden patterns or information from a given dataset. These advanced algorithms have allowed AI-based systems to grow efficiently for the prediction of events without being explicitly designed to do so [11].

**Figure 1:**
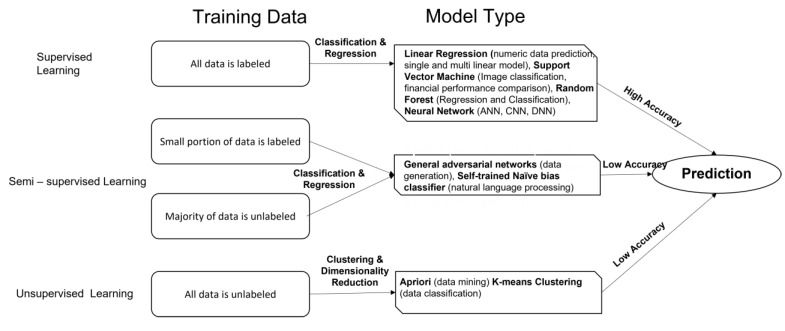
Summary of supervised, semi-supervised, and unsupervised models of machine learning algorithms.

ML is a branch of AI in which the algorithm learns from the data points without being explicitly programmed [12], and is classified into the (1) supervised, (2) semi-supervised, and (3) unsupervised types [13]. In supervised ML algorithms, labelled data points are the input data. The model adjusts the weights to obtain the best fit. Alternatively, in unsupervised learning, the data are not labelled, and the algorithms find the hidden pattern in the dataset for grouping. The semi-supervised one is a method that uses both the supervised and unsupervised principles. It first trains the algorithm on a small, labelled dataset and then, it uses the learning to extract the features from a large, unlabeled dataset. Besides these three classes, there is one additional class of ML that is known as weakly supervised machine learning. This represents the cases when the data in the training set are not adequately labelled as per the ground truth, for instance, due to the excessive cost that is involved in the labelling process. This category can be further subdivided into three classes; (a) incomplete: where the only subset of training data are labelled, (b) inexact: high-level labelling, and (c) inaccurate: the data are not labelled as per the ground truth. The label generation technique can be used on labelled data and unlabeled data. Incomplete supervision is also being used to process the data using human intervention, where the domain expert labels the data. However, it can also use semi-supervised learning to produce generative models. Inexact supervision is performed by CNN and DL techniques, and this has the potential to learn automatically across multiple domains. In inaccurate management, several ensembles are detected as unlabeled and these are checked with the training data and then, the labels are corrected. A summary of the ML classification is shown in Figure 1.

Deep Learning (DL) is the branch of ML that has attracted the most interest in data-centric challenges. In this method, the algorithm is completely independent of human intervention or input, and it devises rules based on the data. Multiple learning layers are deployed in this technique to extract increasingly higher level information/features from the data. DL architecture allows the algorithm to handle big data for the learning process, which assists in the development of robust prediction models. Artificial neural networks (ANN) fall under DL methods, where they can take the data in multiple formats, including images, text, and other unstructured data. An ANN is composed of multiple layers that consist of connected units or nodes that are known as artificial neurons. It starts with the input layer that feeds the data to the network, and later, weighted matrix schemes are applied to different hidden layers. Each hidden layer transforms the input feature by multiplying it with the weight matrix and transferring it to the next consecutive hidden layer. This weighted matrix is optimized using the training and validation dataset to achieve the minimum error in the prediction at the output layer. These weights denote the importance of each feature. A higher weight is assigned to a more important feature. After assigning the weights to the feature data, the ANN applies the transform and activation functions. The transform function uses multiple inputs and converts them into a single output. An active function receives this output, and a non-linear mathematical model is applied to convert the transformed input data into a final prediction.

**Figure 2:**
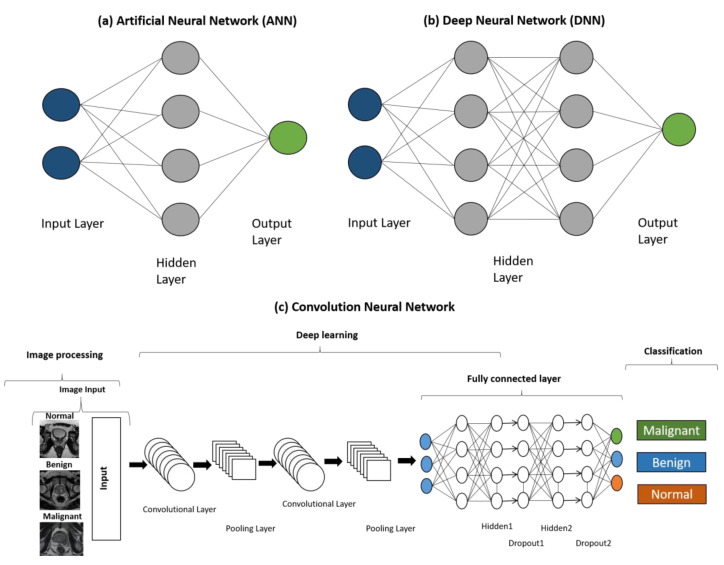
The architecture of neural network machine learning models for data processing and analysis, i.e., (**a**) ANN, (**b**) DNN, and (**c**) CNN methods.

An artificial neural network could be shallow or deep, depending upon the number of hidden layers that are implemented in the network. A DNN, which stands for deep neural network, is an advanced method of ANN where “deep” refers to the depth of the network. This contains multiple hidden layers between the input and output layers. DNN is most suitable one for big data, where the network rigorously trains itself at each layer to reduce the error rate. A modified form of ANN called a convolution neural network (CNN) is intensely used in diagnosis, feeding the diagnostic images to the network and classifying the stage of the disease. It is composed of three layers: (1) the convolution layer: which applies filters to the input image and converts it into a feature map, which is further normalized and resized, (2) the pooling layer: this layer is responsible for the dimensionality reduction, which effectively avoids overfitting, (3) a fully connected layer: this layer is a general characteristic of neural network that takes the input vector and creates an output vector after applying the transform and activation functions. In addition, a specific form of CNN where the last layer is not fully connected is known as a fully convolution network (FCN). U-net is the example of an FCN which does not have a fully connected layer. In the deep neural network architecture, a dropout mechanism is applied to the fully connected layer to reduce the overfitting of the data and produce an unbiased prediction. Figure 2 shows the architecture of distinct types of artificial neural networks.

In recent years, the deployment of AI techniques in the healthcare industry has resulted in significant improvements in PC diagnosis, prognosis, prediction, and disease classification [14]. This review article provides an overview of the AI methods that are being developed and applied successfully for PC diagnosis and treatment.

## 2. Methodology

This study collected articles on the applications of basic and advanced machine learning algorithms that were used for the active surveillance of, the disease staging of, the diagnosis of, and the effective treatment of prostate cancer. Here, the PubMed database was used to collect the articles. It is a freely available data source with multiple options to filter the reports based on structured keywords. In addition, AI is applied in a broad spectrum of domains, and it is highly likely that one would obtain articles from the engineering domain if the search was performed using a non-medical/biological database. Herein, PubMed provided a comprehensive, robust, and highly relevant platform to search for the articles that discuss the application of AI in prostate cancer.

Throughout this investigation, the Preferred Reporting Items for Systematic Review and Meta-Analysis protocols were employed. The keywords string that was formed in this search was: (artificial intelligence) or (machine learning) or (deep neural network) or (deep learning) or (machine vision) or (ai) or (random forest) or (decision tree) or (classification algorithm) or (linear regression) or (modelling) or (support vector) or (decomposable model) or (automatic diagnosis) or (computer aided) or (gaussian mixture modelling) or (natural language progressing) and (prostate cancer) and (review [publication type]).

Initially, these keywords were searched for in multiple phases for the two types of publications that were mentioned in PubMed: (i) the review and (ii) research articles using the advanced search option. Initially, keyword strings were searched for in the title section in the first phase. This implies that keywords should be a part of the title of the review or journal article. This resulted in 41 reviews and 369 research articles. Later, these keywords were searched in the Title/Abstract section to expand the search space. This produced 196 reviews and 2735 research articles. Eventually, these keywords were also searched for in all of the field’s sections; there were 5025 reviews and 46,326 journal articles in the output set. Articles with keywords found in the title were the most relevant for this study as they discussed the principle and application of the AI techniques in prostate cancer. Later, the search output was verified using an intersection exercise. The articles searched from each of the three sections were compared pair-wise for the review and journal articles. The common articles were marked for each pair, title, and title/abstract section (n_1_∩n_2_), the titles were marked with the all fields section (n_1_∩n_3_), and the title/abstracts were marked with the all fields section (n_2_∩n_3_). As a result, it was found that all of the articles that were found using keywords in the title section also included them in the title/abstract section (n_1_∩n_2_ = n_1_). Similarly, all of the articles that were searched for in the title/abstract section were included in the all fields sections (n_1_∩n_3_ = n_1_). By computing the intersection of these three sets (n_1_∩n_2_∩n_3_), we invariably ended up with the set that was selected from the title section only (n1). This showed that the searching technique was consistent as the articles with the keywords contained them in the title of the article. Further, an exclusion criterion was used to screen the articles that were classed as opinion letters, commentary, case reports and surveys, and these were discarded. These exclusion criteria were applied to the 41 journal articles and 369 review articles that were found by the title-based searching.

**Figure 3:**
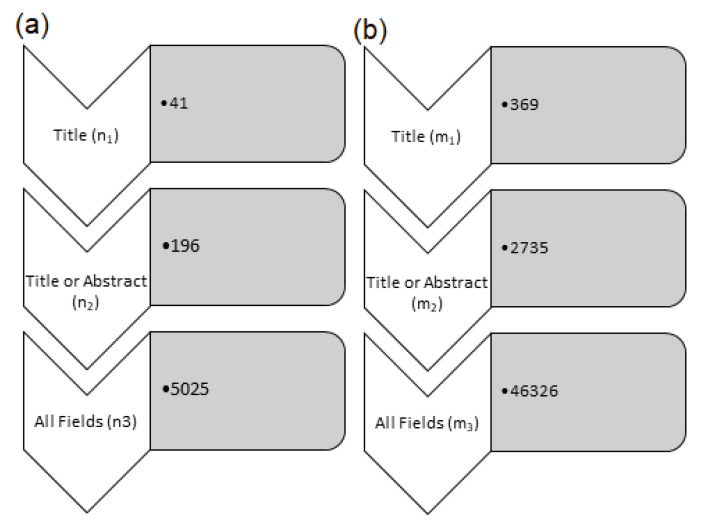
Flow chart of the article selection, i.e., (**a**) review and (**b**) research articles used in the preparation of the present review article.

Moreover, under the review article, there was no entry that was found under the opinion letter, commentary, case report, and survey types. However, the journal article list has 13 commentaries and 1 case report, however, the articles fell under the exclusion classes. Thus, these 14 articles were excluded from the present study. Finally, the selected papers were evaluated for their direct relevance to the objective of this review article (application of AI-machine learning in prostate cancer diagnosis and treatment). In the first phase, their abstracts and introductions were reviewed to collect the necessary content that was presented in the introduction section of this review article. Later, the most relevant articles were closely examined for their results and conclusions to present their data in the latter sections. Figure 3 illustrates a flowchart of the data collection that was used in this review study.

**Figure 4:**
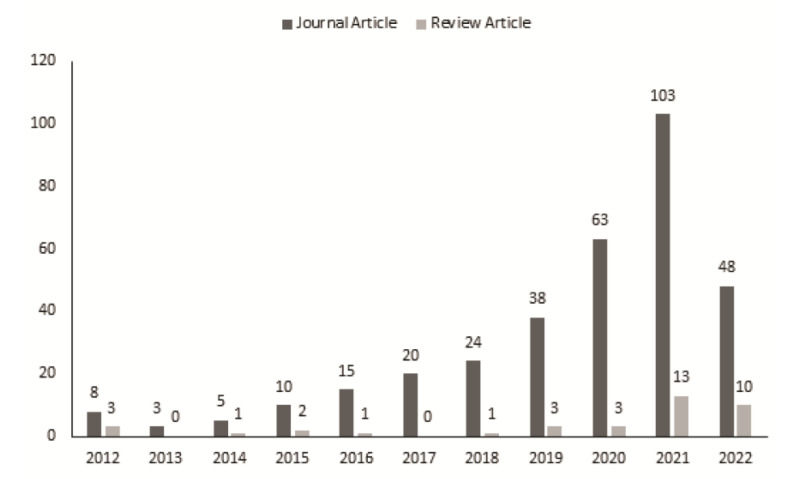
Published articles count for the last 10 years for both review and journal articles.

Additionally, the studies referred to in the screened articles were also included in this review article. Later, these articles were evaluated according to the year that they were published. Figure 4 shows that the number of machine-learning-based studies on prostate cancer has increased significantly in recent years. The number of publications, particularly in the last full year (2021), has been relatively high for both the reviews and journal articles.

## 3. Results

### 3.1. Application of AI in Prostate Cancer Diagnosis

#### 3.1.1. AI in Biopsy-Based Detection of Prostate Cancer

A biopsy is a procedure that involves the separation of a sample of cells or a piece of tissue from the body for laboratory analysis. This is applicable when the patient is symptomatic or when a physician has identified a substantial concern. A biopsy test can detect the presence of cancerous cells in a patients’ sample [15]. The classification of the biopsy methods that are used to diagnose cancer is discussed in Table 1.

Depending on the method, a histological examination of a biopsy sample may include the chemical modification or freezing of it before the section preparation of it for its examination under a microscope. However, the complete process of examination is a manual, extensive process and requires pronounced precision. In contrast, due to a shortage of urological pathologists, the existing human resources are insufficient to handle the high volume of biopsies that are collected for a PC examination. This leads to a condition where the examination could be conducted partially or fully using an AI system.

Gleason grading is one of the systems that is primarily used in prostate cancer detection and treatment planning [20]. It is determined by urological pathologists examining the prostate biopsies. Table 2 shows the classification of the Gleason grading system with the pathological histological system [21]. Herein, a low score indicates slow-progressing prostate cancer, which aids in identifying the severity of the condition. A typical area has a score that is below 3, whereas a malignant area has a score that is greater than or equal to 3. A sample can exhibit varied Gleason scores.

The Gleason score for the needle biopsy correlates with pathological variables, including the margin status of radical prostatectomy specimens, prostate specific antigen levels, tumor volume, and related molecular markers [22]. The human eye cannot easily detect the errors in the scoring method due to the ink on the slides, the cutting artefacts, and there being rare cancer subtypes. Moreover, the Gleason score in several cases underestimates the severity of the disease. In this context, Nagpal et al. developed a DL-based model to improve the Gleason score for the prostate cancer slides that are obtained during prostatectomies [23]. In this model, 112 million image patches of 1226 slides annotated by the pathologists were used and tested on 331 slides from 331 patients. When the results were compared to the diagnostics and grading by 29 expert urologic pathologists, the accuracy (mean) that was reported was 0.61 on the validation dataset. However, the DL algorithms provided a higher accuracy rate of 0.70 [23]. This method indicated the direct application of DL in classifying the images and compared them with the human eye detection method.

Moreover, this also showed the scalability of training. An algorithm could be trained on a large dataset (112 million), and it could store the information perpetually. Furthermore, this dataset could be increased, which can directly improve the learning of an algorithm. In contrast, the human expert systems can only store a limited set of information which can result in the wrong classification and quantification of unseen cases.

Later, to achieve a pathologist level of accuracy for Gleason grading, an AI system based on the DL method was developed [24]. This designed system had a training dataset of 5209 hematoxylin and eosin-stained digitized biopsies from 1243 patients, while 550 biopsies were used to evaluate the model method. In addition, 160 random samples from the test set were used for the manual evaluation of biopsies to compare the AI-based system with the observations of the pathologists [24].

An ML-based method with a cascade approach has been developed using the Gleason grading method to differentiate the needle biopsies of prostate cancer into multiple classes to provide a more differentiated classification of the cells [25]. An AI-based algorithm was designed for automated Gleason grading with high sensitivity and accuracy. The tool used 698 biopsies from 174 patients for training, and it was tested on a set of 37 biopsies from 21 patients. The results showed that the algorithm was 100% sensitive to the validation dataset [26]. However, the size of the dataset is the decisive factor in the training and testing of any AI-based computing approach. Thus, multiple cross-validations (3-fold or 5-fold) are required to attain a higher prediction accuracy for the new dataset. In developing ML algorithms, the dataset’s diversity must also be accounted for. Of note, cross-validation is a technique that is used to minimize the overfitting in the prediction model. It indirectly estimates the true performance of the prediction model. If a model shows a high accuracy in the cross-validation experiment, then the prediction of this model would have higher reliability factor due to the unbiased nature of the model. Considering the sensitivity and specificity of it in the light of there being similar scores between the training and test samples can also indicate the robustness of the prediction algorithm.

Convolution neural networks (CNN) are used to enhance the accuracy of the Gleason pattern and the Gleason grading-based classification of the histopathological samples from a prostate cancer patient. The algorithm was designed to improve the PC diagnosis accuracy, as the expert pathologist reported numerous errors in manual grading method. The algorithm was trained on 96 tissue specimens of digitized slides from the biopsies of 38 patients. However, the study overestimated the accuracy with fewer training datasets [27]. The CNN method is designated to be the most appropriate algorithm for image classification. However, the speed of the CNN-based training is a challenging concern due to the slower calculation in the maxpool layer. This study used a dataset of 96 images, which is considered to be a small dataset, and the CNN model can be easily trained. In a large set of biopsy images, a more robust computing system is required to perform training using the CNN method. In another similar study, an AI-based algorithm was designed to predict the PC grading and quantification at a higher degree of precision. The system was developed using a training dataset of 838 digitized biopsies and a test dataset of 162 digitized biopsies from the whole image. Here, the dataset was larger than it was in the earlier study, which indicates higher confidence and reproducibility in the prediction. A panel of three pathologists was employed to evaluate the system using the manual and AI-assisted grading of the biopsies. It was concluded that AI assistance helped the pathologist to make observations with a reduced analysis time. In addition, implementing the AI method reduced the inconsistency in the final results [28].

#### 3.1.2. Artificial Intelligence in MRI-Guided PC Detection

An MRI is a medical imaging technique that employs a magnetic field and radio waves that are generated by a computer to capture comprehensive images of the organs and tissues of the body. This approach is incredibly useful in identifying prostate cancer (PC) or other abnormalities in a variety of internal organs, and it addresses the limitation of producing a negative biopsy report for actual positive cases.

Schnall et al. attempted to fuse pathology and imaging in 1991, and they had marginal success in correlating the radiographic and pathological markers [29]. Ward et al. investigated image-driven specimens with strand-shaped fiducial markers in 2012 to obtain a digital registration of the images from histology tests and in vivo MRIs [30]. Since 2014, Litjens et al. have devised a technique to classify MRI images and further characterize them using different machine learning models, which served as the foundation for the more recently designed and implemented approaches (analyzing computerized images of the prostate and the pixels for fundamental image processing) [31].

Prostate segmentation is critical for detecting its deformable capsule, which has applications in prostate fusion biopsy and brachytherapy. These data are captured using MRI and transrectal ultrasonography (TRUS). Gaur et al. demonstrated in a multi-institutional study that implemented AI algorithms for detection purposes that improved the sensitivity of the images by 78% when they were used it with PI-RADS (Prostate Imaging-Reporting and Data System) v.2 (version 2) [32]. In this study, AI algorithms resulted in an efficient performance in the transition zone (TZ) compared to the whole prostate and peripheral zone. This technique showed a sensitivity of 84% with automated detection compared to this being 67% when MRI was used alone [33].

Multiple major studies have shown that MRI is an effective method for differentiating clinically significant PCs from non-significant PCs. It emerged as an alternative method to transrectal ultrasonography-guided biopsy to direct the pathologist to the accurate site for excising the tissue samples [34,35,36]. The MRI method is mostly used one to detect the cancer stage by recording the image data that show the spread of the cancer outside the prostate. These images have highly granular information that is often particularly challenging to interpret. This process can be automated with increased accuracy by using an AI-based machine-learning algorithm.

Artificial intelligence (AI) has been proposed to assist in diagnosing and identifying prostate cancer (PC) as the MRI outcome has early significance in the diagnostic process. The aggressive and non-aggressive forms of PC are critical to distinguish between due to their highly differential prognosis. Before a biopsy, the European Association of Urology (EAU) has recommended using multiparametric MRI, which is a crucial step in the diagnostic process that has been followed in several studies [37]. Moreover, the predictions made by these ML programs should assist the doctors in making the final decision instead of completely ruling out the doctor’s interpretation.

Considering the rise in prostate cancer cases, there is a need for computer-based methods using AI for an improved and fast assessment of the prostate MRI data [38]. An AI algorithm that was based on cascade deep learning was developed for the enhanced detection and bi-parametric classification of prostate MRIs, and it applied the Prostate Imaging Reporting and Data System (PI-RADS) score. The algorithm used a dataset of 1390 samples that were obtained at 3 Tesla for the model training, testing, and validation. All of the samples were also accessed by a radiologist. The algorithm was trained for automated detection and image segmentation using a 3D U-Net-based residual network architecture. The algorithm was found to have a good detection and classification performance in detecting cancer suspicious lesions [39]. The resolution of these MRI images is critical for machine learning approaches. However, the cost and infrastructure that are required to set up a high-field MRI scanner are the limitations to generating high-resolution images for many cases. Algorithms need to be developed more frequently in specific cancer domains to improve the resolution of the MRI scan images. This could allow us to use a large set of cleaned MRI scans in a machine learning algorithm. Later, radiomics-based methods were employed using T2-weighted images (T2W) that were more accurate than the apparent diffusing coefficient (ADC) in the MRI data, as the ADC that was associated with the Gleason score for categorizing the disease stage appeared to function more efficiently [40]. Recently, researchers showed that innovative radiomics using high-B value diffusion weighted imaging (CHB-DWI) and ADC modalities outperformed a clinical heuristics-driven method for prostate cancer detection [41]. Aldoj and his co-workers showed another DL-based technique [42], a CNN employing multiple 3D combinations (ADC, DWI, T2-weighted pictures) with an area under the curve (AUC) parameter of 0.91 with 81.2% sensitivity and 90.5% specificity when it was compared to a radiologist using PI-RADS v2 [43]. Further, the deep CNN approach was designed to analyze and classify mpMRI prostate lesion images from the ImageNet dataset. The technique was found to be reliable and accurate in the classification of malignant lesions from non-cancerous tissues [44]. AI has also been used recently to design, train, and validate a CNN that can decide whether to carry on with biparametric MRIs or dynamic contrast-enhanced sequences (DCE) in mpMRIs. The study was performed on 300 prostate MRIs, and the method was found to be 94.4% sensitive and 68.8% specific for accessing the needs of the DCE sequences. This research may aid in avoiding DCE-MRI when it is not necessary, hence preventing the DCE-induced negative effects [45]. Deep learning is the most suitable choice for the cases with a large set of features where CNN has proven its higher performance for image classification including in MRI scan images [46]. Several CNN architectures have been designed to improve the accuracy of the prediction. Alexnet, VGG, ResNet, and Googlenet are a few important and widely used CNN architectures. ML algorithms are also being applied for the image reconstruction. Recently, convolutional recurrent neural networks have begun to be applied for this image reconstruction. Data augmentation is required for medical imaging, including for PC-MRI scan images. This would generate a dataset that can be optimally used in the CNN network (VGG) for an improved PC diagnosis, detection, and segmentation.

#### 3.1.3. Artificial Intelligence in Transrectal Ultrasound-Guided Biopsy-Based PC Detection

A transrectal ultrasound biopsy (TRUS) is used for the detailed imaging of the prostate gland and the surrounding tissues. In a TRUS biopsy, in the patient’s rectum, a device called a transducer generates high-frequency sound waves. These waves travel through the body and are reflected back to the transducer after hitting the internal structures, such as the prostate gland. The transducer converts the returning sound waves into an image called a sonogram. These sonogram images help to decide the location of the biopsy needles and where to take the tissue samples from [47]. Before the advent of techniques like MRI imaging, TRUS was the gold standard for guiding the location of the prostate cancer needle biopsies. An increasing number of prostate cancer cases makes it difficult for pathologists to keep up the pace with the diagnostic procedures. Therefore, the ANN-based AI system was designed to assist the clinicians’ in making decisions. The model was repeatedly trained with variables like the digital images from TRUS, prostate-specific antigen (PSA) levels, and age. The validation was performed using a TRUS image output, and the system could efficiently differentiate between the malignant and non-malignant prostate tissues [48].

On the other hand, TRUS has poor detection and staging accuracy in prostate cancer as it produces low-contrast images. However, when it is compared to MRI, TRUS provides the advantages of being less costly, more convenient in the office, and by it providing a real-time snapshot. TRUS has an overall tumor (T) staging accuracy of between 80% and 95% when it is compared that which is between 75% and 85% for MRIs, except for the T4 stage when TRUS only provides anterior tumor images. Furthermore, when it is compared to MRI, TRUS has a limitation of having a remarkably high degree of operator dependency.

**Figure 5:**
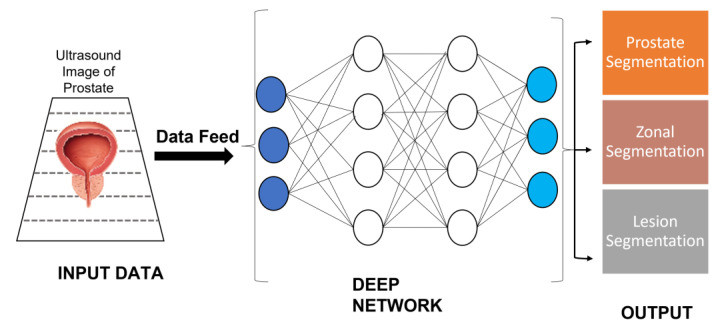
Application of deep neural network to classify segmentation in prostate cancer using TRUS image. Herein, input data: data collected from the medical examination of patients and healthy individuals. Data feed: sending structured and processed data to the machine learning model. Deep network: the architecture of machine learning model. Output: prediction for a different types of prostate cancer segmentation.

The advancement in prostate cancer diagnosis has developed MRI-TRUS fusion-guided needle biopsies to efficiently predict prostate cancer [49,50]. The technique is time-consuming and laborious, so AI has been introduced to automate the process to reduce the burden on clinicians. A dataset including TRUS pictures was obtained from three institutions utilizing an Aixplorer (Supersonic Imagine, Aix-en-Provence, France) ultrasound scanner, an iU22 (Philips Healthcare, Bothell, WA, USA), a Pro Focus 2202a (BK Medical), respectively. The datasets that were used in the study obtained 436 images of 181 men. This deep learning method was designed to perform an automated segmentation of the TRUS images for MRI. The model was evaluated based on its median accuracy (98%), Hausdorff distance (3.0 mm), and the Jaccard index (0.93). The pixel-wise accuracies for the zonal segmentation of the peripheral and transition zones were reported to be 97 and 98%, respectively. The technique has increased the speed of MRI-TRUS fusion-guided biopsies to target cancerous lesions [51]. Figure 5 shows the application of TRUS to the deep neural network for predicting prostate segmentation, zonal segmentation, and lesion segmentation in PC cases. Here, the MRI-TRUS fusion-guided biopsies approach assists doctors in detecting hidden cancers that other prostate biopsies can miss. It can execute targeted biopsies by focusing on the problematic regions, directly utilizing advanced MRI/ultrasound fused images. This method has proven to be particularly effective for men who had previously negative biopsies. However, it may also assist in detecting aggressive malignancies in patients who have had no previous biopsy.

In addition to the studies that are mentioned in this section, a summary of AI/machine learning algorithms used in PC diagnosis is provided in Table 3. The studies mentioned in Table 3 are similar in terms of their mathematical modelling and ML algorithms when they are compared to the techniques that are discussed in this section. However, there are differences in the dataset collection, preparation, and processing processes.

#### 3.1.4. Artificial Intelligence in 3D Pathology Based PC Detection

The pathological study of the biopsies and surgically removed tissues is critical for the disease’s diagnosis and its characterization. This detailed examination of the tissue’s morphological and molecular properties is critical in deciding which therapies are best for patients. For many malignancies, the biopsy-determined grade of the illness is used to stratify the patients for clinical care, which might result in drastically divergent therapy pathways [60,61].

The recent advancement of the use of whole slide imaging (WSI) scanners by a number of hospitals and healthcare institutions that have begun digitizing their entire pathology workflows, combined with rapid increases in the computational power, has resulted in the proliferation of digital pathology approaches in oncology and other areas [62,63,64,65]. Kaneko et al. introduced a unique pilot AI method for the 3D prediction of PCa in their study. The integration of multiparametric MR-US image data with fusion biopsy trajectory-proven pathology data was used to train the AI. The AI prediction was much higher than the radiologist’s reading was, and it was in concordance with the data of the clinically significant cancer (CSCa) center when it was using the robot-assisted radical prostatectomy (RARP) specimens (83 percent vs. 54 percent, *p* = 0.036). The AI predicted CSCa volumes that were more accurate (r = 0.90, *p* = 0.001) than the radiologist’s readings were [66].

Currently, prostate cancer care relies heavily on two-dimensional (2D) histopathology [67], which involves formalin fixing and paraffin embedding (FFPE) a set of core-needle biopsies to allow for thin sections to be cut, mounted on glass slides, and stained for their microscopic study. The Gleason grading method is used to measure the aggressiveness of the cancer, which is purely based on a visual interpretation of the prostate gland morphology as seen on a few histology slides (thin 2D tissue slices) of only a “sample” of around 1% of the whole biopsy. The prostate cancer Gleason grading is linked with substantial interobserver variability and is only marginally connected with the outcomes, particularly in individuals with intermediate-grade prostate cancer [68,69,70]. This contributes to the under-treatment of patients with aggressive cancer [71], which leads to avoidable metastasis and death [72], and the overtreatment of patients with indolent cancer [73], which can result in serious side effects such as incontinence and impotence [74].

Furthermore, Xie et al. created a method for the non-destructive 3D pathology and computational analysis of the entire prostate biopsies which are tagged using a speedy and affordable fluorescent equivalent of conventional hematoxylin and eosin (H&E) staining. This analysis is based on the interpretable glandular characteristics and is made possible by the advancement of image translation-assisted 3D segmentation (ITAS3D). ITAS3D is a generalizable deep learning-based technique for volumetrically segmenting the tissue microstructures in an annotation-free and objective (biomarker-based) way without immunolabeling them. They photographed 300 ex vivo samples from 50 stored radical prostatectomy cases, of which 118 included a malignancy, to demonstrate the translational utility of a computational 3D pathology method vs. a computational 2D pathology approach. Based on the clinical, biochemical recurrence outcomes, the 3D glandular characteristics of the cancer biopsies outperformed the similar 2D features in a risk assessment of the individuals with low- to intermediate-risk prostate cancer [75].

#### 3.1.5. Artificial Intelligence in Genomics-Based and Proteomics-Based PC Detection

There is a rising interest in the genomics and proteomics of PC and how mutations in the PC genome might influence the individual evolution of PC [76]. Prostate-specific antigen (PSA) level testing has aided in the diagnosis and prognosis of prostate cancer [77,78].

Over the last decade, an avalanche of biomarkers have been found and are being used in clinical assays [79,80,81,82,83,84,85,86,87,88]. While many of these biomarkers have been researched and defined according to the function of each test, there is no typical overlap among all of these assays, and there is no completely ideal list of biomarkers that are used to predict the diagnosis and prognosis of PC. Consequently, it is essential to identify and analyze new biomarkers of clinical significance in a meaningful and dependable manner.

Thus, the ANN technique under AI can be beneficial in assessing these biomarkers. According to a study, Ki67 is a significant indicator of survival and illness progression [89]. Green et al. built an ANN model to validate Ki67 gene expression while comparing it to another probable candidate in DLX2 [90]. Ki67 and DLX2 were also significant predictors of future metastases in the univariate analysis. However, only 6.8% of the individuals with prostate cancer had a substantial Ki67 expression. As a result, this study demonstrated that these two indicators could only be used to select the patients for them to receive focused treatment. The co-expression of multiple genes needs to be studied to build a strong prediction platform.

Moreover, the most suitable approach is multi-omics, where genomic, transcriptomic, and metabolomic data must be combined and fed into the machine learning algorithms. Here, the interpretability of the prediction model is also critical, while in a deep neural network model, it behaves as a black box. Basic prediction models always have high interpretability but limited accuracy in their prediction making. This indicates a trade-off between accuracy and interpretability. Another limitation or point of concern for the application of a fully connected dense network is overfitting. However, overfitting can be controlled using regularization techniques, but it comes with the cost of a high computing power.

Biological phenotypes are the direct or indirect reflection of the genomic sequence. The biological sequence data are large scale, and thus, a deep learning method is suitable to study these data and determine the association of the sequence patterns with the phenotypic properties of cancer. Here, the sequence data can provide the early detection of cancerous tissue. The application of deep learning to genomic sequence data is often termed genomic deep learning (GDL). It establishes the relationship between the sequence variation and the cancer-associated traits. It is shown that prediction performance is improved by developing a specific ML model for different cancer types [91]. An open tool was designed to apply the deep learning algorithms directly to the biological sequence. This algorithm uses CNN and LSTM to predict the secondary structure, subcellular localization, and binding of peptides to the MHC Class II molecules. Additionally, this can be sourced from GitHub and modified for PC detection [92]. NGS (next-generation sequencing) data have been used with the system biology information, and an ML model was created to characterize the tumour and tumour type [93]. The DNA sequence of the patient is used in the machine learning model to classify the cancer patient [94].

**Figure 6:**
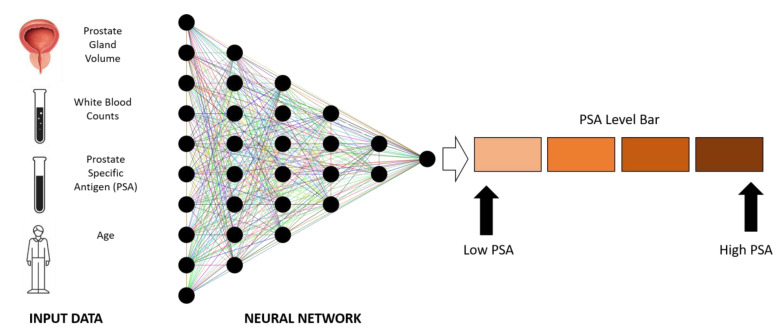
Usage of prostate-specific antigens (PSA) and other patient information to predict the chance of receiving a positive prostate biopsy.

In addition to gene expression, proteomics data can also be valuable in detecting possible biomarkers. Kim et al. used a distinctive technique to find new potential proteomic markers for prostate cancer by integrating targeted proteomics with computational biology [95]. The investigation was initiated with 133 differentially expressed proteins in a cohort of 74 patients tested with synthetic peptides [96]. Later, using these candidates, a machine-learning methodology was used to construct the clinical prediction models [97,98]. This result indicates that computationally driven proteomics can uncover new non-invasive biomarkers. Several research studies have shown the ability of AI ANNs to facilitate more effective biomarker identification and validation, which may aid prostate cancer monitoring. Figure 6 shows the application of PSA and other supportive patient data to predict the chance of a positive prostate biopsy using an ANN. The prostate-specific antigen (PSA) test is a blood test that detects the number of PSA molecules that are present in the blood. This test can be helpful in the detection of prostate cancer, the monitoring of its treatment, and the evaluation of its recurrence. There is always a detectable level of prostate-specific antigen (PSA) in the blood samples of men, however, a low level of PSA is considered normal, while an elevated level of PSA can be an indicator of prostate cancer.

#### 3.1.6. Artificial Intelligence in CT Scan-Based PC Detection

An artificial intelligence (AI) algorithm created by Australian researchers can detect the early symptoms of prostate cancer by analysing normal computed tomography (CT) images. The study was performed at RMIT University and by St Vincent’s Hospital Melbourne researchers. The AI system was developed by examining asymptomatic individuals with and without prostate cancer CT scans to identify the disease’s characteristics [99]. As per the early studies, CT scans are highly acceptable data points for diagnosing bone and joint disorders, but the radiologists had difficulties recognising prostate cancer in the CT scan images. This approach is not recommended for a normal cancer examination because of its high radiation doses, which may have long-term effects. Furthermore, AI technology may be used to screen for cancer in men whose abdomen or pelvis were being diagnosed with other complications. In this study, the AI programme was trained to seek for illness signs in a range of scans and to detect the area of examination in the image, thus eliminating the need to manually trim the input images. The algorithm’s performance was assessed by comparing it to the results of the professional radiologists using cross-validation techniques on a dataset of 571 CT images of the abdomen and pelvic areas. The AI algorithm resulted in a better outcome and assisted in identifying malignant growths in a short time (~seconds). In addition, the AI approach improves with each scanned image, learning to interpret the scans from various machines to detect even the smallest anomalies.

Dr Mark Page from St. Vincent’s hospital in Melbourne mentioned that this technology could allow healthcare workers to detect prostate cancer early. The patients with critical conditions that had CT scans might be tested for prostate cancer simultaneously, and its early identification by this technology could significantly improve their prognosis [99]. Here, the early diagnosis of prostate cancer is an essential factor for the patients. Even in the case of a false positive prediction using CT scan-based ML predictions, the patients can undergo other tests where the diagnosis can be re-confirmed. However, a false negative in the final prediction could lead to a problematic stage. Thus, these algorithms must be trained on diverse CT scan data to reduce the false negative prediction rates. The patient must go through radiation therapy, which is very expensive. The contrast between the prostate and the tissues around it is not strong enough in CT images to make it easy to separate the prostate from the other tissues. Simultaneously, CT scans can help to detect prostate cancer’s spread in bone tissue and determine whether prostate brachytherapy is required.

### 3.2. Artificial Intelligence in PC Treatment

The treatment decision process for newly diagnosed prostate cancer patients is complicated and demands a shared decision-making process as various treatment options are available, including surveillance. In the PROTECT study, 1643 men were randomly assigned to radical prostatectomy, or radiation and active monitoring [100]. It was reported that the cancer-specific mortality rate was minimal, independent of randomized therapy at a 10-year median follow-up, and all of the therapies were statistically equivalent. The study suggested that when surgery and radiation were used as treatment options, they greatly reduced the development of cancer and metastases. Still, these treatment processes also brought higher comorbidities that impacted their quality of life. An informed decision is based on various elements, such as the biological description of the illness, the treatment outcome, adverse effects, and, most importantly, the patient’s preferences based on the patient-specific data.

Electronic medical records and clinical registries are already collecting huge quantities of data that are easily accessible for data mining [101]. However, these registries must undergo an adequate analysis and interpretation to provide the therapeutically relevant advantages to the patients. Historically, statistical models have served this purpose. However, they cannot analyze the data with high dimensions and cannot dynamically adapt to the augmentation of new data points. Although the data from clinical registries assist the doctors in making data-driven choices, the patients have few opportunities to gain access to these registries actively and further, be informed about future decisions. As clinicians are expected to handle massive volumes of data ranging from macro-level physiology and behavior to laboratory investigations, and increasingly, “-omic” data, artificial intelligence (AI) is the best solution to use these data to build a prediction model. The ability of AI to manage this complexity has outperformed the methods of human accuracy and management. It helps the doctors to understand the patient’s condition genuinely.

**Figure 7:**
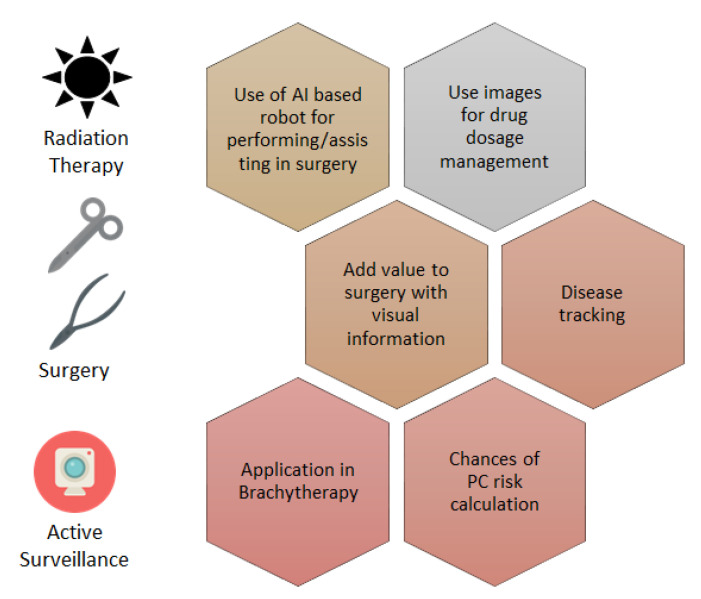
Summary of AI direct application and assistance in PC treatment.

Auffenberg et al. demonstrated a prediction program where the patient’s medical information was used to build a prediction model using random forest machine learning techniques that can further assist in making treatment decisions [102]. In this study, the data registry included 7543 men who were diagnosed with prostate cancer, 45% of whom were treated with radical prostatectomy, 17% of whom were treated with radiotherapy, and 30% of them were treated with surveillance, 6% of them were treated with an androgen deprivation treatment, and 2% of them were treated with a watchful waiting treatment. In addition, the data were divided into the train and test subgroups using a 2:1 randomized split that was classified by training location. Overall, the customized model was significantly accurate, which showed the area under the curve (AUC) of 0.81 for a classification. This further also concluded that age is the most relevant variable that influences the patient’s treatment decisions, while the number of positive cores also affects the treatment process, and this is followed by the Gleason score. The random forest method is less interpretable than the decision tree is, but it is more accurate as it is composed of multiple decision trees. As in this study, a limited number of numerical data points were fed to the model. Even basic algorithms such as the random forest can produce an acceptable prediction. However, a deep network can be explored if an extensive collection of patient records is available. In general, multiple ML methods need to be tested, and their AUCs must be compared before the model is finalized. The characteristic of the data determines the suitability of data basic or advanced ML models. Figure 7 summarizes the application of AI in PC treatments.

### 3.3. Recent Advancements and Future Aspects

The diagnosis and treatment of prostate cancer demand the wide application of deep learning methods. Recently, publications have been published that include several DL implementations that help urologists to diagnose PC at different stages. Table 4 presents an overview of the several models that have been employed in recent research, along with their evaluation matrix (area under curve score, AUC), which indicates their accuracy in identifying prostate cancer. The small dataset and the absence of a federated learning strategy were identified as the two major limitations of these computer-aided detection techniques. Federated learning models can be used to enhance the process of collecting and sharing data for research objectives. As a result of adequate training, increasing the sample size may improve the performance of multilayer DL models. Increased the sample size allows the neural networks with more hidden layers and nodes to extract a broad spectrum of feature sets and avoid early overfitting. An increase in the variables utilized to identify prostate cancer can also improve the performance of a neural network model.

### 3.4. Available Codes and Programs

A few studies made the source code public, and these are available at the github. prostatecancer.ai (accessed on 30 June 2022) is the platform that provides an AI model in the web browser for the computer-assisted detection, diagnosis, and prognosis. Their codes are available at https://github.com/Tesseract-MI/prostatecancer.ai (accessed on 31 June 2022). As discussed, bpMRI images are helpful in PC detection. A source code for Hierarchical Probabilistic 3D U-Net is available at https://github.com/DIAGNijmegen/prostateMR_3D-CAD-csPCa (accessed on 31 June 2022) for public use. In the genetic data, sigminer prediction developed a model to predict the cancer subtypes based on the mutational signature. The source code is available at https://github.com/ShixiangWang/sigminer.prediction (accessed on 30 June 2022). A deep learning network using CNN was developed to detect the glandular cells in the digitized biopsies, and the source code was made available at the github https://github.com/alvarillo89/Glands-detection (accessed on 1 July 2022).

## 4. Conclusions

This review article applied an organized search approach using the search string of multiple keywords in PubMed to collect the relevant reports. The methodology mentioned in this study depends on the keyword formation and their combination using a logical operator. PubMed has multiple filter criteria to customize the search. The search was oriented towards the application of AI in prostate cancer. There has been continuous rigorous discussion and controversy around the application of artificial intelligence in the medical/treatment domain. However, technological advancements in AI algorithms and data generation have recently made significant progress and contributed mainly to the diagnostic and treatment domain. AI has the potential to extract valuable features from a large dataset and establish sophisticated relationships between the prediction variable and other known parameters. However, the manual management and prediction of large-scale datasets are not feasible. Furthermore, diagnostic and risk assessments are essential for active surveillance studies and early prostate cancer detection. AI has reduced the subjectivity of the outcome and made it possible to conduct tests with fewer resources while improving the overall competence and precision. The FDA has authorized the use of AI in detecting prostate cancer. The risk of false negatives is reported less often in this method as it is performed by doctors and pathologists considering laboratory studies, patients’ history, and other relevant clinical information. AI-assisted diagnostics in PC biopsies can improve the quality of the outcome and cut down on the price and time that are involved. However, AI is not used to replace human expertise in detecting PC, but to reduce the chance of missing actual positive cases.

## Figures and Tables

**Table 1:** Classification of biopsy methods used in the detection of PC.

S. No.	Biopsy Techniques	Summary
1	Needle Biopsy	This method of biopsy inserts a needle into the skin for collecting cells from a suspicious area. This process is also known as a percutaneous tissue biopsy by doctors [16].
2	Endoscopic biopsy	Endoscopy is a procedure in which medical staffs use a flexible and thin tube (endoscope) with a light at the terminal to examine structures within the body. Further, special instruments are inserted into the tube to collect a tiny tissue sample to analyze [17].
3	Skin biopsy	A skin biopsy collects cells from the surface of the skin. It is mainly used to identify skin diseases, including melanoma. The type of cancer that is detected and the extent of the suspicious cells will determine the sort of skin biopsy that is experienced by the patient [18].
4	Bone marrow biopsy	This biopsy method is mainly used after the findings of blood tests or if the doctors propose a malignancy that affects the bone marrow [19].
5	Surgical biopsy	A surgical biopsy may be prescribed if other biopsy procedures are ineffective or if the results of the initial tests have been inconclusive.

**Table 2:** Classification of Gleason grading system with histopathological PC samples.

Grade and Gleason Score	Type	Pattern	Size
1, Score ≤ 6	Benign	Single glands with sharp boundaries that are well defined and consistent.	Medium
2, Score 3 + 4 = 7	Benign	Single glands are widely apart and the tumor’s boundaries are not clearly defined; it is less well confined.	Medium
3, Score 4 + 3 = 7	Malignant	Masses that are single, separated, spherical, irregular, or larger and have a cribriform or papillary pattern.	Small to large
4, Score 4 + 4 or 3 + 5 or 5 + 3 = 8	Malignant	Fused gland tumour with predominantly pale cells and no architecture.	Small to medium
5, Score 4 + 5 or 5 + 4 = 9 and 5 + 5 = 10	Malignant	Tumors and cords of comedo cancer, solid sheets and no gland formation.	Small

**Table 3:** Summary of studies that mentioned the application of AI/machine learning in diagnosing the prostate cancer.

S. No.	Summary	Date Accessed	References
1.	This study shows that a higher grade PC is related to increased epithelial volume and lower stromal and lumen map volumes measured by hybrid multi-dimensional MRI, thus making this a potential approach in predicting aggressive PC.	29 August 2022	[52]
2.	TRUS-Bx remains useful in PC diagnosis when it is paired with mpMRI. This study showed the application of AI algorithms in prostate gland segmentation, lesion identification, and classification using mpMRI and TRUS-Bx, reducing interreader variability and minimising the possible lack of competence of less experienced radiologists.	29 August 2022	[53]
3.	This diagnostic study suggested that an AI-based assistive tool can increase the accuracy, speed, and consistency of pathologists’ assessment of prostate biopsy samples. The relatively high number of samples and pathologists involved in this study allowed for a thorough examination of the advantages of an AI-based tool for the contemporaneous assessment of prostate biopsies, as well as insights into potential risks associated with overreliance.	29 August 2022	[54]
4.	As per the findings showed in this study, 18F-1007-PSMA PET-based radiomics features with 40–50% standardized uptake value (SUV) max exhibited the most robust predictive ability for evaluating numerous PC biological characteristics. Radiomics properties, when compared to a single PSA model, may give significant benefits in predicting the biological aspects of PC based on the support vector machine. The 50% SUVmax model had the most powerful predictive performance in trained (AUC, 0.82) and tested cohorts for predicting Gleason score (GS) (AUC, 0.80). The 40% SUVmax model has the most significant expected performance for extracapsular extension (ECE) (AUC, 0.77). In terms of vascular invasion (VI), the 50% SUVmax model performed the best (AUC 0.74).	29 August 2022	[55]
5.	In this study, artificial intelligence ultrasound of the prostate (AIUSP) detected the PC (49.5%) when it was compared to transrectal ultrasound (TRUS)-guided 12-core systematic biopsy (34.60%) and mpMRI (35.80%). Clinically significant PC (csPC) detection rate in AIUSP group was 32.30%, which was compared to TRUS-SB (26.3%) and mpMRI (23.1%) groups. The overall biopsy core positive rate in the TRUS-SB (11.0%) and mpMRI (12.7%) groups was substantially lower than it was in the AIUSP group (22.7%).	29 August 2022	[56]
6.	The weighted low-rank matrix restoration algorithm (RLRE) algorithm was used to de-noise MRI images in this study to identify PC from benign prostatic hyperplasia (BPH) and to evaluate the diagnostic impact of MRI images with varied sequences. The findings showed that the RLRE algorithm might increase MRI images’ presentation effect and resolution. However, RLRE algorithm-based MRI images of the DCE sequence were more useful in the differential diagnosis of PC and BPH, thus facilitating disease therapy.	29 August 2022	[57]
7.	The objective of this study was to extend artificial intelligence (AI) models that detect cancer in the prostate that extends to areas outside of it. Herein, by merging different models with image post-processing procedures and clinical judgement criteria, an autonomous strategy was developed to detect cancer spread outside the prostate barrier using prostate MRI images.	29 August 2022	[58]
8.	This study observed that a deep learning-based algorithm using only H&E-stained digital slides can correctly predict ERG rearrangement status in most cases of prostatic adenocarcinoma. An artificial intelligence-based model could eliminate the need for extra tumour tissue to be used in ancillary studies to look for ERG gene rearrangement in prostatic adenocarcinoma. All of the models had comparable receiver operating characteristic (ROC) curves with area under the curve (AUC) values ranging from 0.82 to 0.85. These models’ sensitivity and specificity were 0.75 and 0.83, respectively.	29 August 2022	[59]

**Table 4:** Summary of dataset used in several studies with their respective performance on PC detection.

S. NO.	Dataset	Method	AUC	References
1.	The bpMRI of 1513 including 73 patients 2 consecutive bpMRI scans with clinical variables (PSA, PSA density, and age)	Deep learning algorithm	0.86	[103]
2.	Trans-rectal prostate biopsy of 109 patients	Random forest Neural network Ctree Support vector machine	0.83 0.74 0.74 0.72	[104]
3.	Dataset of 551 patient including age, BMI, hypertension, diabetes, total PSA (tPSA), free PSA (fPSA), the ratio of serum fPSA to tPSA (f/tPSA), prostate volume (PV), PSA density (PSAD), neutrophil-to-lymphocyte ratio (NLR), and pathology reports of prostate biopsy	Tpsa logisticregression Multivariate logistic regression Decision tree Random forest Support vector machine	0.84 0.91 0.92 1.00 0.88	[105]
4.	dataset of 315 patients available with preoperative T2WI, DWI, ADCMR images. Also, Trus-guided 12-needle puncture was performed within 3 months after MRI and provided P504S and P63 status	Random forest Gradient boosting Decision tree Logistic regression AdaBoost K-nearest neighbours.	0.92 0.91 0.89 0.89 0.89	[106]
5.	356 patients undergoing transrectal ultrasound-guided prostate biopsy	Logistic regression Decision tree classifier Dense neural network	0.80 0.78 0.94	[107]
6.	103 patients with mpMRI scan, PI-RADS V2 score was 4/5 and Prostatic biopsy results confirmed prostatic hyperplasia or PC	R-logistic R-SVM R-AdaBoost	0.93 0.84 0.73	[108]
7.	438 men with metastatic prostate cancer	Gradient boosting machine Model1 Model2 Model3 Model4 Model5 Model6	0.76 0.73 0.86 0.82 0.79 0.79	[109]
